# A small molecule iCDM-34 identified by in silico screening suppresses HBV DNA through activation of aryl hydrocarbon receptor

**DOI:** 10.1038/s41420-023-01755-w

**Published:** 2023-12-22

**Authors:** Yutaka Furutani, Yoshinori Hirano, Mariko Toguchi, Shoko Higuchi, Xian-Yang Qin, Kaori Yanaka, Yumi Sato-Shiozaki, Nobuaki Takahashi, Marina Sakai, Pornparn Kongpracha, Takehiro Suzuki, Naoshi Dohmae, Mutsuko Kukimoto-Niino, Mikako Shirouzu, Shushi Nagamori, Harukazu Suzuki, Kaoru Kobayashi, Takahiro Masaki, Hiroo Koyama, Kazuma Sekiba, Motoyuki Otsuka, Kazuhiko Koike, Michinori Kohara, Soichi Kojima, Hideaki Kakeya, Tomokazu Matsuura

**Affiliations:** 1 Department of Laboratory Medicine, The Jikei University School of Medicine, 3-25-8 Nishi-shimbashi, Minato-ku, Tokyo, 105-8461 Japan; 2 Liver Cancer Prevention Research Unit, RIKEN Cluster for Pioneering Research, 2-1 Hirosawa, Wako, Saitama, 351-0198 Japan; 3 Biomolecular Characterization Unit RIKEN Center for Sustainable Resource Science (CSRS), RIKEN, 2-1 Hirosawa, Wako, Saitama, 351-0198 Japan; 4 Center for SI Medical Research, The Jikei University School of Medicine, 3-25-8 Nishi-shimbashi, Minato-ku, Tokyo, 105-8471 Japan; 5 Department of Mechanical Engineering, Keio University, Yokohama, Kanagawa 223-8522 Japan; 6 Laboratory for Computational Molecular Design, RIKEN Center for Biosystems Dynamics Research (BDR), 6-2-3 Furuedai, Suita, Osaka, 565-0874 Japan; 7 Laboratory for Cellular Function Conversion Technology, RIKEN Center for Integrative Medical Sciences, 1-7-22 Suehiro-cho, Tsurumi-ku, Yokohama, Kanagawa 230-0045 Japan; 8 Department of System Chemotherapy and Molecular Sciences, Division of Medicinal Frontier Sciences, Graduate School of Pharmaceutical Sciences, Kyoto University, Sakyo-ku, Kyoto, 606-8501 Japan; 9 Laboratory for Protein Functional and Structural Biology, RIKEN Center for Biosystems Dynamics Research, Tsurumi-ku, Yokohama, Kanagawa 230-0045 Japan; 10 Laboratory of Biopharmaceutics, Meiji Pharmaceutical University, 2-522-1 Noshio, Kiyose, Tokyo, 204-8588 Japan; 11 Drug Discovery Chemistry Platform Unit, RIKEN Center for Sustainable Resource Science, 2-1 Hirosawa, Wako, Saitama, 351-0198 Japan; 12 Department of Gastroenterology, Graduate School of Medicine, University of Tokyo, Tokyo, 113-8655 Japan; 13 Department of Gastroenterology and Hepatology, Okayama University Graduate School of Medicine, Dentistry, and Pharmaceutical Sciences, Okayama, 700-8558 Japan; 14 Department of Microbiology and Cell Biology, Tokyo Metropolitan Institute of Medical Science, Kamikitazawa, Setagaya-ku, Tokyo, 156-8506 Japan; 15 Sasaki Institute Shonan Medical Examination Center, 10-4 Takarachou, Hiratsuka-shi, Kanagawa 254-0034 Japan

**Keywords:** Target identification, Screening, Small molecules

## Abstract

IFN-alpha have been reported to suppress hepatitis B virus (HBV) cccDNA via APOBEC3 cytidine deaminase activity through interferon signaling. To develop a novel anti-HBV drug for a functional cure, we performed in silico screening of the binding compounds fitting the steric structure of the IFN-alpha-binding pocket in IFNAR2. We identified 37 compounds and named them in silico cccDNA modulator (iCDM)-1–37. We found that iCDM-34, a new small molecule with a pyrazole moiety, showed anti-HCV and anti-HBV activities. We measured the anti-HBV activity of iCDM-34 dependent on or independent of entecavir (ETV). iCDM-34 suppressed HBV DNA, pgRNA, HBsAg, and HBeAg, and also clearly exhibited additive inhibitory effects on the suppression of HBV DNA with ETV. We confirmed metabolic stability of iCDM-34 was stable in human liver microsomal fraction. Furthermore, anti-HBV activity in human hepatocyte-chimeric mice revealed that iCDM-34 was not effective as a single reagent, but when combined with ETV, it suppressed HBV DNA compared to ETV alone. Phosphoproteome and Western blotting analysis showed that iCDM-34 did not activate IFN-signaling. The transcriptome analysis of interferon-stimulated genes revealed no increase in expression, whereas downstream factors of aryl hydrocarbon receptor (AhR) showed increased levels of the expression. CDK1/2 and phospho-SAMHD1 levels decreased under iCDM-34 treatment. In addition, AhR knockdown inhibited anti-HCV activity of iCDM-34 in HCV replicon cells. These results suggest that iCDM-34 decreases the phosphorylation of SAMHD1 through CDK1/2, and suppresses HCV replicon RNA, HBV DNA, and pgRNA formation.

## Introduction

HBV, a major cause of HCC, infects approximately 296 million people around the world [1]. Currently, entecavir (ETV) and tenofovir, which are nucleotide/nucleoside analogs that inhibit reverse transcription, are mainly used to suppress viral replication [2]. However, as there is a possibility of viral reactivation and carcinogenesis, it is desirable to develop a therapeutic agent that can function as a complete cure.

Interferon (IFN) induces interferon-stimulated genes (ISGs) in host cells, which suppress HBV replication, but is only effective in approximately 30% of patients [3]. Other drugs such as entry inhibitors, capsid formation inhibitors, Toll-like receptor agonists, RNA interference, and genome editing reagents have been developed before [4]. However, there is no therapeutic agent that can completely eliminate covalently closed circular DNA (cccDNA) of HBV. Pegylated IFN-α2a (pegIFN-α2a; PEGASYS) is commonly used as a therapeutic agent for the treatment of HBV infection. Although it is effective in only about 30% of patients, a mechanism of cccDNA degradation via APOBEC3A and ISG20 has been proposed [5], suggesting the possibility of a complete cure. However, PEGASYS is known to cause side effects such as fever and malaise. Direct-acting antiviral drugs such as nucleotide/nucleoside analogs and capsid formation inhibitors can inhibit the replication of HBV, but they cannot eliminate cccDNA and do not lead to complete cure of the infection [4]. Therefore, the development of drugs that work on host factors and suppress cccDNA is awaited.

Small compounds that act on host cells and exhibit anti-HBV activity, such as IFN-α/β receptor 2 (IFNAR2) agonist CDM-3008, Toll-like receptor agonists, and farnesoid X receptor agonists, have been reported [6–9]. These agents are candidates for a functional or complete cure. Chemical screening has shown that CDM-3008 (RO8191) is a small molecule compound that acts as an IFNAR2 agonist and suppresses HCV [10]. We have also showed that CDM-3008 has anti-HBV and anti-SARS-CoV-2 activities [11]. In addition, CDM-3008 has an additive inhibitory effect with ETV [7]. Knockdown experiments have shown that CDM-3008 acts only on IFNAR2 and does not require IFNAR1 [7, 10]. In addition, CDM-3008 is a small chemical compound that can be administered orally, while IFN is administrated by an injection that requires hospital visits. To develop orally available drugs for HBV infection treatment, we conducted an in silico screening of small chemical compounds based on the pocket information of the binding site of IFN-α within the IFNAR2 extracellular region. As a result, identified compounds that activate aryl hydrocarbon receptor (AhR) and exhibit anti-HBV activity, while the compounds did not activate IFN signaling.

## Results

**Fig. 1:**
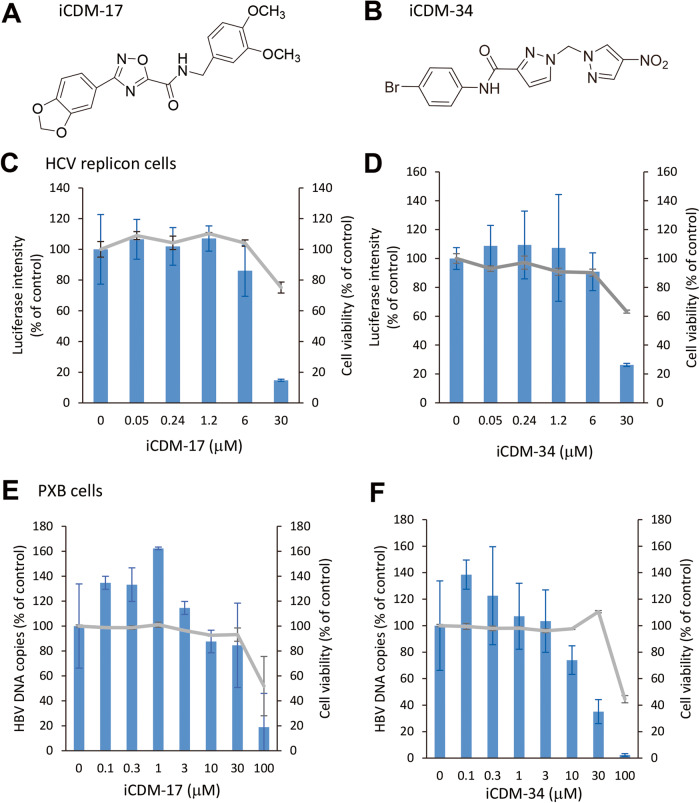
Analysis of anti-HCV and anti-HBV activities of iCDM-17 and -34. Chemical structure of iCDM-17 (**A**) and iCDM-34 (**B**). HCV replicon cells were treated with 0.05–30 µM iCDM-17 (**C**) and iCDM-34 (**D**) for 3 days, and luciferase intensity (blue bars) and cell viability (gray lines) were measured. Primary human hepatocytes were infected with HBV genotype C and cultured for 28 days, and then treated with 0.03–100 µM iCDM-17 (**E**) and iCDM-34 (**F**) for 7 days. Cell viability was measured using the XTT assay kit (gray lines), and cellular HBV DNA levels were measured by qPCR (blue bars). Error bars indicate SD (*n* = 3).

To choose the IFNAR2-binding candidate compounds, we performed the docking simulation in 2 steps. Firstly, top 1000 compounds were selected from 300,000 ranked by GoldScore (GOLD). Secondly, we identified 37 candidate compounds from 1000 ranked by combination score of GoldScore (GOLD), GlideScore (Glide), and S score (MOE). We analyzed IFN-like activity of 37 candidate compounds using HCV replicon cells and identified two compounds and named them iCDM-17 (Fig. 1A, C) and iCDM-34 (Fig. 1B, D) as these compounds were detected from in silico studies using docking simulations. To measure their anti-HBV activity, HBV-infected PXB cells were treated with serially diluted iCDM-17 and -34. iCDM-17 and -34 were found to suppress cellular HBV DNA levels at half maximal inhibitory concentrations of 56.6 µM and 19.7 µM in a dose-dependent manner, respectively (Fig. 1E, F). Moreover, the anti-HBV activity of iCDM-34 was more potent than that of iCDM-17.

**Fig. 2:**
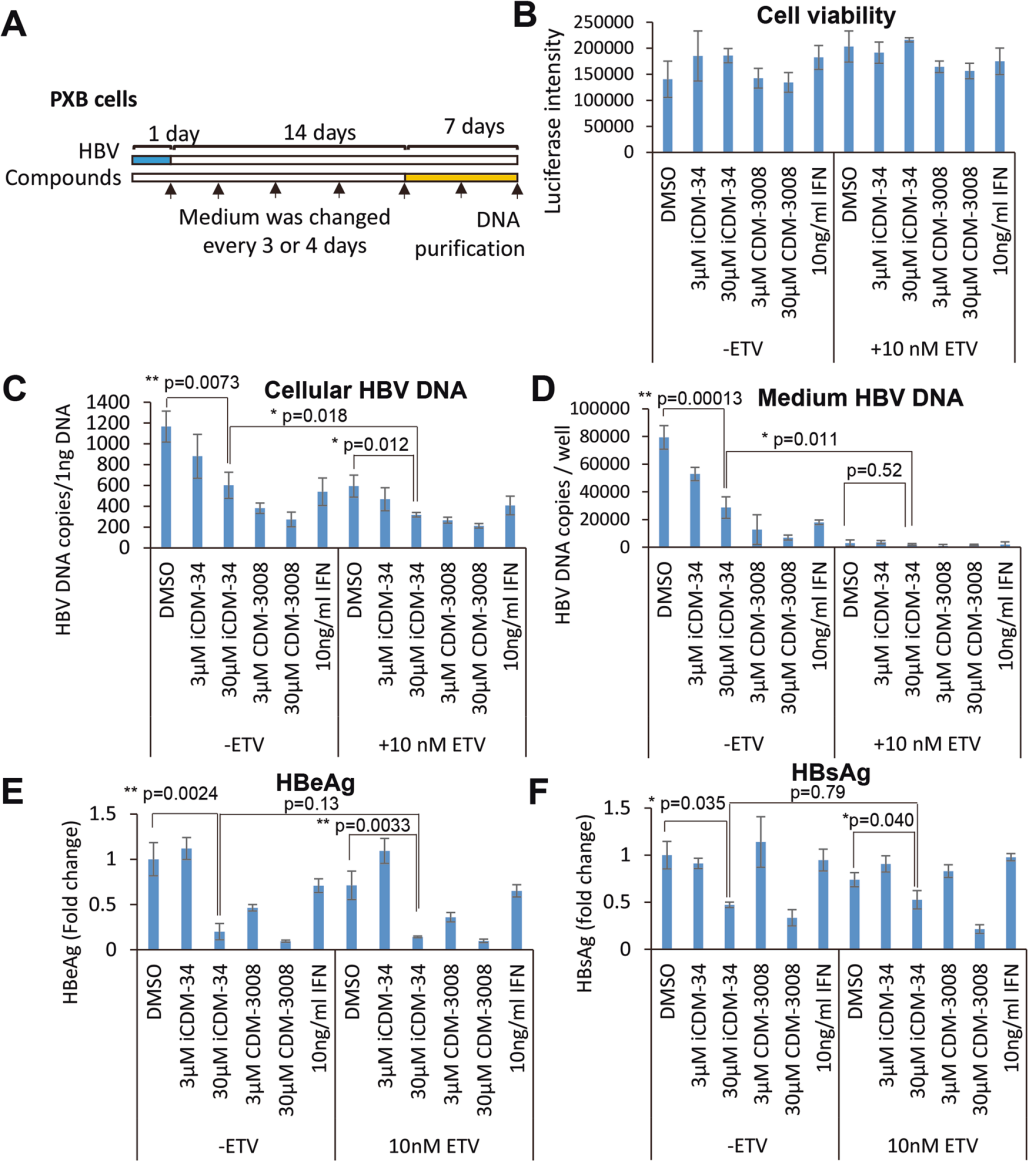
Anti-HBV activity of iCDM-34 and additive effects with entecavir (ETV) in primary cultured human hepatocytes. **A** The schematic experimental design of the anti-HBV activity analysis. Primary cultured human hepatocytes (PXB cells) were infected with HBV genotype C for 1 day (blue) and cultured for 14 days, and the cells were then treated with 3 and 30 μM iCDM-34 and CDM-3008 and 10 ng/mL pegylated interferon (pegIFN)-α2a for 7 days (orange). The black arrows show the time points of medium changes. After the treatments, cell viability was measured and cellular DNA was purified. **B** Cell viability was measured by RealTime-Glo MT Cell Viability Assay and shown as % of DMSO control. **C** Cellular HBV DNA levels in 1 ng DNA were measured. **D** Medium HBV DNA levels were measured in the medium. **E**, **F** HBsAg and HBeAg levels in the medium were measured by ELISA. Error bars indicate SD (*n* = 3). *p < 0.05 and **p < 0.01 (two-tailed *t* test).

We selected iCDM-34 with a pyrazole moiety for further analysis and investigated its anti-HBV activities and additive effects with ETV using HBV-infected primary human hepatocytes. The cells were treated with 3 and 30 µM iCDM-34 and CDM-3008 and 10 ng/mL pegIFN-α2a with or without 10 nM ETV for 7 days (Fig. 2A). These treatments showed no cytotoxicity (Fig. 2B). Cellular HBV DNA decreased with iCDM-34 and CDM-3008 treatments in a dose-dependent manner. In addition, additive effects of ETV were confirmed in combination treatments with iCDM-34 and CDM-3008 (Fig. 2C). Furthermore, 30 µM iCDM-34 treatment inhibited medium HBV DNA, HBsAg, and HBeAg with showing a additive effect with ETV (Fig. 2D–F).

**Fig. 3:**
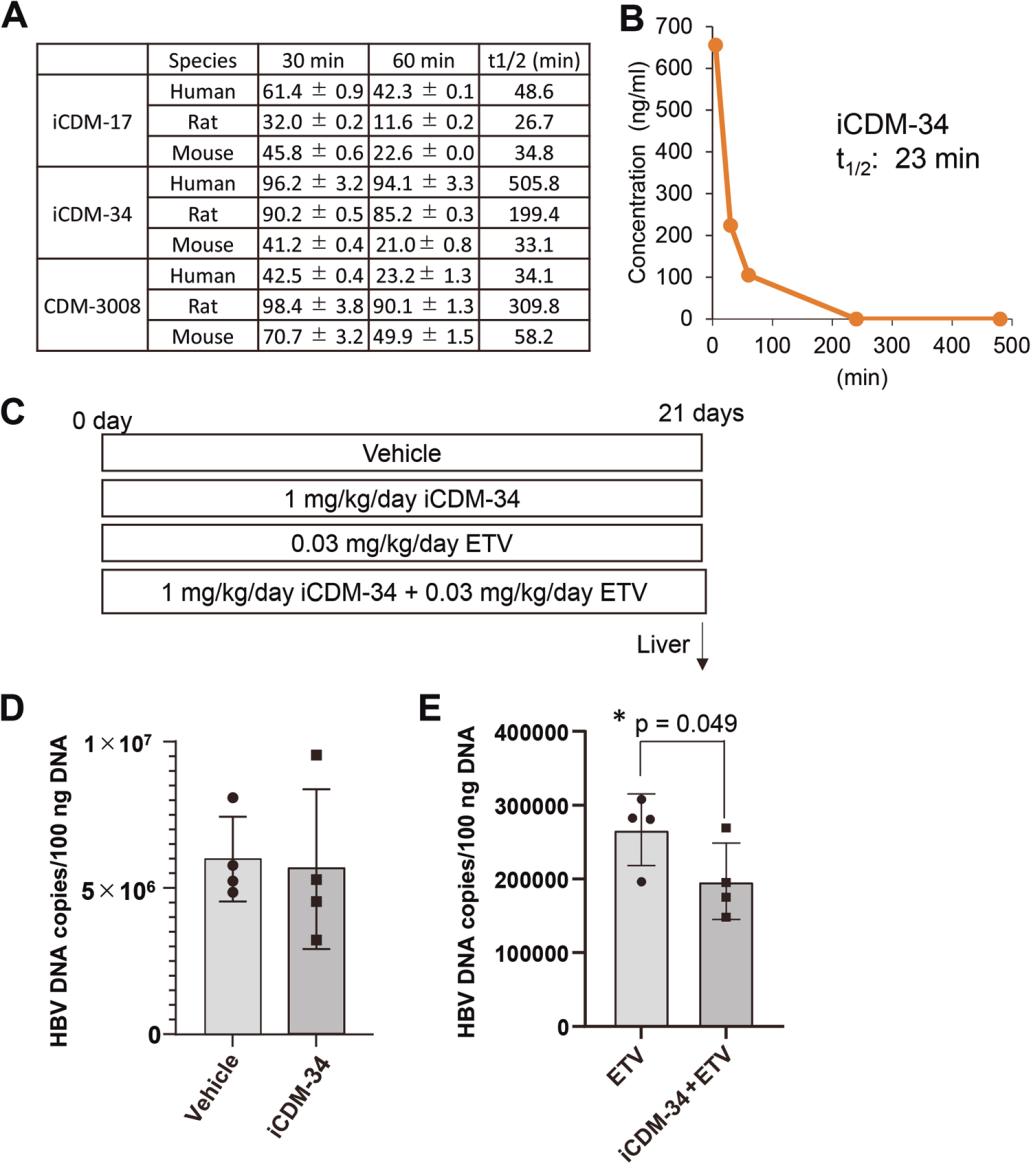
Metabolic analysis of iCDM-34 and anti-HBV activity of iCDM-34 in human hepatocyte-chimeric mice. **A** Metabolic analysis of iCDM-17, iCDM-34, and CDM-3008 in human, rat, and mouse liver microsomal fractions. **B** Metabolic analysis of iCDM-34 in vivo. iCDM-34 was injected into human hepatocyte-chimeric mouse, and then iCDM-34 concentration in the blood was measured at 5, 30, 60, 240, 480 min after the injection (*n* = 1). **C** iCDM-34 (1 mg/kg, i.v.) was injected with or without ETV (0.03 mg/kg, p.o.) once a day. After 21 days of injections, DNA was purified from the liver, and then HBV DNA (**D**, **E**) levels with or without ETV were measured by qPCR. The data are presented as means ± SD (*n* = 4). * *p* < 0.05 in one-sided student’s *t* test.

To analyze the pharmacokinetics of iCDM-34, we performed metabolic experiments using human, rat, and mouse microsomal fractions and found that iCDM-34 was stable in human liver microsomes at *t*_1/2_ = 505.8 min (Fig. 3A). In human hepatocyte-chimeric mice, iCDM-34 showed a metabolic rate of 23 min (Fig. 3B). Finally, anti-HBV activity was measured in human hepatocyte-chimeric mice with i.v. administration of iCDM-34 as a single agent or in combination with ETV (Fig. 3C). As a result, iCDM-34 alone did not reduce HBV DNA in the liver, but the combination of iCDM-34 and ETV drastically suppressed the amount of HBV DNA in the liver compared with ETV alone (Fig. 3D, E).

**Fig. 4:**
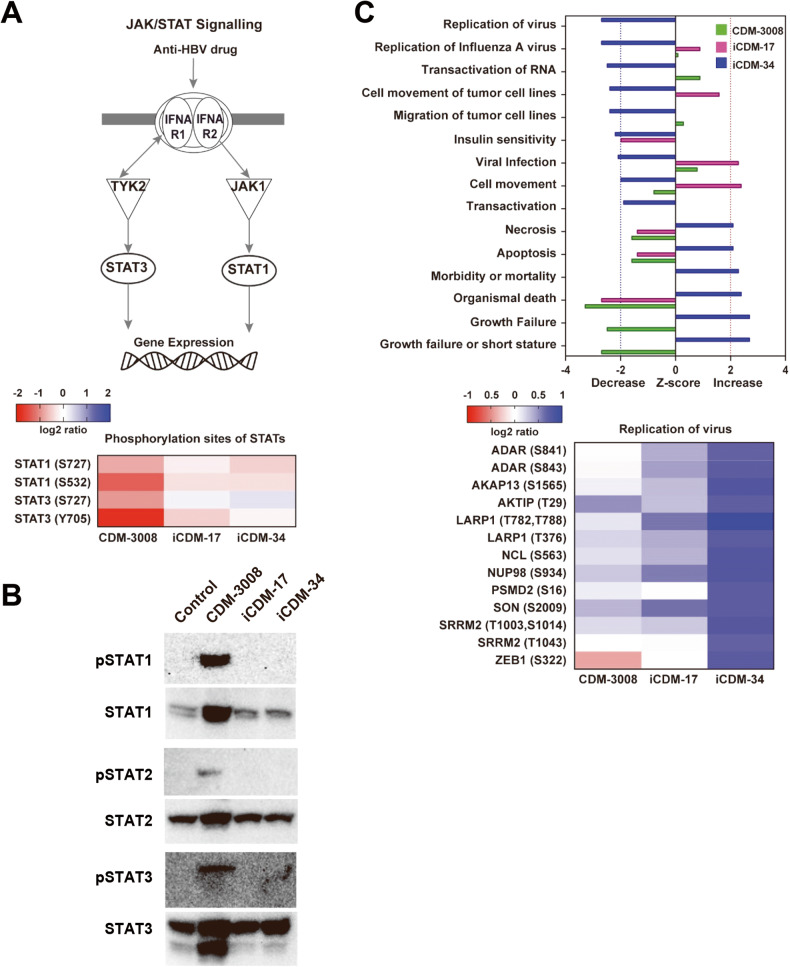
Proteomic analysis of phosphorylated proteins induced by iCDM-34 treatment. **A** The effect of anti-hepatitis B virus drug; CDM-3008, iCDM-17, iCDM-34 on JAK/STAT signaling. The heatmap illustrates the phosphorylation state of STAT1 and STAT3, known phosphoproteins stimulated by drug; CDM-3008. The colors represent the log2 ratio of drug versus DMSO. **B** The effect of CDM-3008, iCDM-17, iCDM-34 on JAK/STAT signaling was confirmed by Western blotting. Phosphorylation of STAT1, STAT2, STAT3 was analyzed after the 24 h of treatments with 30 μM CDM-3008, iCDM-17, and iCDM-34. **C** Top 15 biological functions altered by iCDM-34 treatment. The bars are corresponding to z-scores; the positive or negative z-score values indicate a function that was predicted to be increased or decreased by CDM-3008 (Green), iCDM-17 (Pink) and iCDM-34 (Blue). The dash lines represent the z-score = 2 (red dash) or = −2 (blue dash). The z-score greater than 2 or smaller than -2 can be considered significant altered. All the biological functions are significant (*p*-value ≤ 0.05). The lower panel heatmap shows the phosphoproteins involved in “Replication of virus”, which were specifically decreased by iCDM-34. The colors represent the log2 ratio of drug versus DMSO.

To analyze signal transduction induced by iCDM-34, phosphoproteome analysis was performed wherein iCDM-34-treated samples showed inhibition of an HCV replication. To analyze the IFN signaling pathway, we examined the phosphorylation of signal transducer and activator of transcription 1 (STAT1) and STAT3. The phosphorylation of STAT1 and STAT3 activated by CDM-3008, an IFNAR2 agonist, decreased with iCDM-17 and -34 treatment, with a greater decrease in the phosphorylation of STAT3 (S727) (Fig. 4A). Western blotting analysis of phospho-STAT1, STAT2, STAT3 also showed that phosphorylation of STAT1, STAT2, and STAT3 were induced by CDM-3008, but not induced by iCDM-17 and iCDM-34 (Fig. 4B and Supplemental Fig. 1). The phosphorylation of proteins involved in replication of the virus was highly suppressed by iCDM-34, indicating it possessed a different phosphorylation profile from that of CDM-3008 and iCDM-17 (Fig. 4C). These results suggest that iCDM-34 does not induce IFN signaling pathway and induces different characteristic signal pathways compared to iCDM-17 and CDM-3008.

**Fig. 5:**
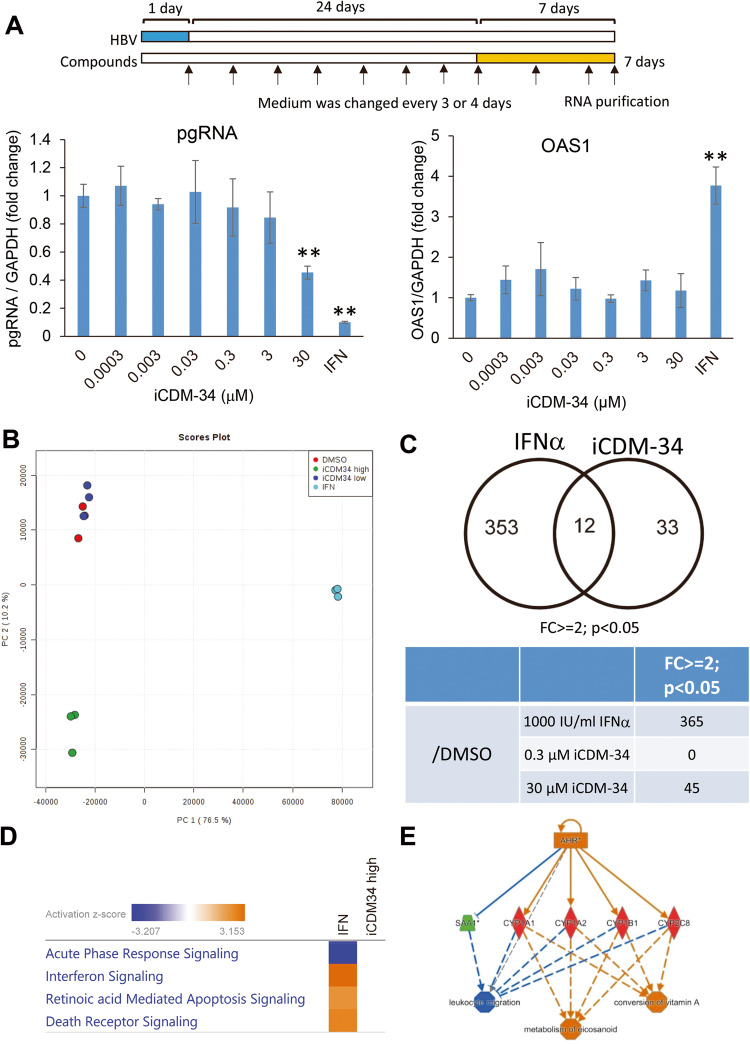
Analysis of iCDM-specific signaling pathway on gene expression in HBV-infected PXB cells. **A** Schematic of the experimental design of HBV infection and IFN-α and iCDM-34 treatments prior to microarray analysis. PXB cells were infected with HBV genotype C for 1 day (blue) and cultured for 24 days. The cells were then treated with 1000 IU/mL IFN-α or 0.0003–30 μM iCDM-34 for 7 days (orange). The black arrows show the times at which the medium was changed. After the treatments, *pgRNA* and *OAS1* mRNA expression levels were measured by qPCR. Error bars indicate SD (*n* = 3). **p < 0.01 (two-tailed *t* test). **B** Score plot of principal component analysis on microarray-based gene expression profiling of PXB cells treated with vehicle control DMSO, 0.3 μM and 30 μM iCDM-34, or 1,000 IU/mL IFN-α for 7 days (upper panel in **C**). Comparison of differentially expressed genes in PXB cells with treatment with 1,000 IU/mL IFN-α or 30 μM iCDM-34 for 7 days with an absolute fold-change > 2 and *p* < 0.05 relative to the vehicle control DMSO (lower panel in **C**). **D** Canonical pathway analysis of differentially expressed genes induced by 1,000 IU/mL IFN-α or 30 μM iCDM-34 treatment using the knowledge-based functional analysis software Ingenuity Pathways Analysis (IPA). **E** Upstream regulator analysis of AhR using IPA with differentially expressed genes specifically induced by 30 μM iCDM-34 treatment.

To analyze the molecular mechanism of anti-HBV activity induced by iCDM-34 treatment, HBV-infected PXB cells were treated with serially diluted iCDM-34 for 7 days. *Pregenome RNA* (*pgRNA*) expression level significantly decreased with 30 µM iCDM-34 treatment. However, there was no increase in the expression of *OAS1*, an ISG (Fig. 5A). Based on the chemical structure of iCDM-34, we hypothesized that iCDM-34 is partially homologous to nitazoxanide, and thus could inhibit the interaction between HBx and DNA binding protein 1. However, iCDM-34 did not inhibit the interaction (data not shown). To elucidate the mechanism of action of iCDM-34, we performed transcriptome analysis of PXB cells treated with 0.3 and 30 µM iCDM-34 and 1,000 IU/mL pegylated IFN-α. The gene expression patterns were different between the 30 µM iCDM-34 and 1,000 IU/mL IFN-α treatment groups (Fig. 5B). The differentially expressed genes in PXB cells after treatment with 1,000 IU/mL IFN-α and 30 μM iCDM-34 for 7 days were compared. The 45 genes induced by iCDM-34 treatment were identified with an absolute fold-change > 2 and *p* < 0.05 relative to the vehicle control DMSO (Fig. 5C). Among the 45 genes, 12 were found to overlap with IFN-α-induced genes (Fig. 5C).

**Fig. 6:**
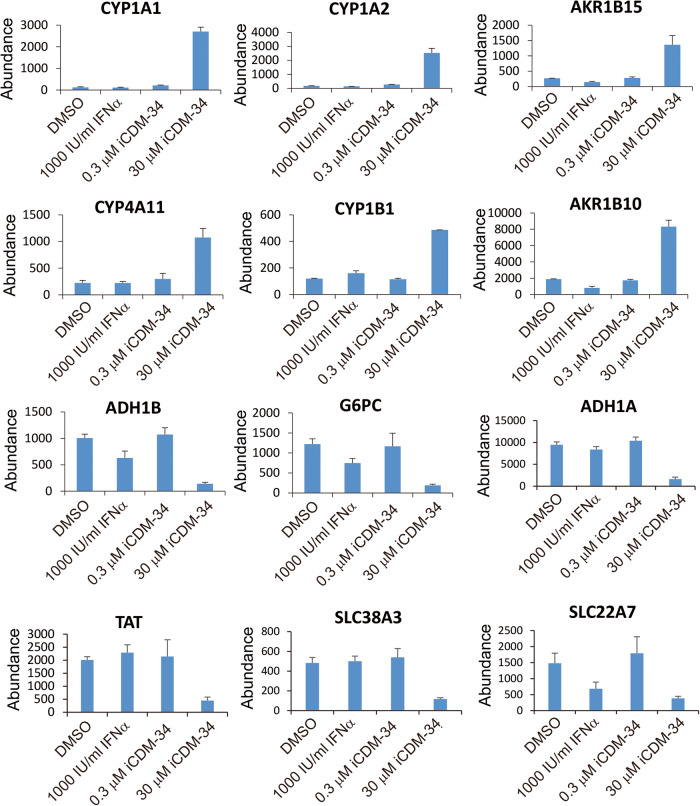
Expression levels of genes associated with AhR signaling. HBV-infected PXB cells were treated with 1000 IU/mL IFN-α and 0.3, 30 µM iCDM-34, and mRNA expression levels of AhR-related genes are summarized from the expression analysis data using microarray. Error bars indicate SD (*n* = 3).

**Fig. 7:**
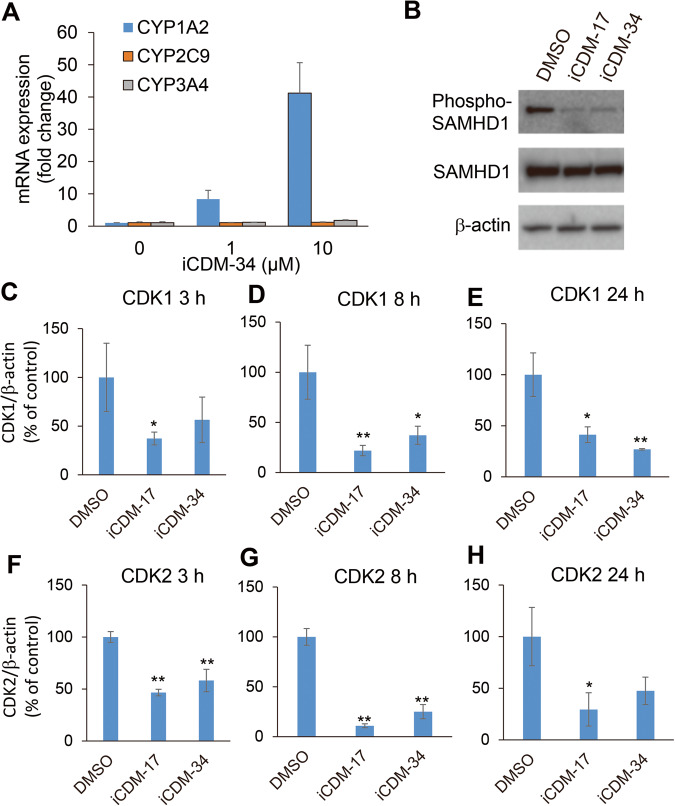
Functional analysis of iCDM-34-mediated induction of anti-HBV activity. **A** Activation of AhR signaling pathway in 3D cultured FLC-4 cells. 3D cultured FLC-4 cells were treated with 0, 1, 10 μM iCDM-34 for 1 day. *CYP1A2* (blue bars), *CYP2C9* (orange bars), and *CYP3A4* (gray bars) mRNA expression levels were measured by quantitative PCR. Error bars indicate SD (*n* = 3). **B** HepG2-NTCP-C4 cells were treated with 30 μM iCDM-17 and iCDM-34 for 1 day. Expression levels of phospho-SAMHD1, SAMHD1, and β-actin were analyzed by Western blotting. **C**–**H** HepG2-NTCP-C4 cells were treated with 30 μM iCDM-17 and iCDM-34 for 3, 8, 24 h. Expression levels of CDK1 (**C**–**E**) and CDK2 (**F**–**H**) mRNA were analyzed by qPCR. Error bars indicate SD (*n* = 3). The data are presented as means ± SD. *p < 0.05 and **p < 0.01 in two-tailed student’s *t* test.

IPA analysis of canonical pathway showed that iCDM-34 did not activate the IFN signal (Fig. 5D). We performed upstream regulator analysis using the 45 genes and found that AhR was predicted as an upstream regulator for iCDM-34-induced genes (Fig. 5E). *CYP1A1*, *CYP1A2*, *CYP4A11*, *CYP1B1*, *AKR1B15*, and *AKR1B10* were upregulated, while the expression of *ADH1B*, *G6PC*, *ADH1A*, *TAT*, *SLC38A3*, and *SLC22A7* was suppressed with iCDM-34 treatment (Fig. 6). Furthermore, treatment of 3D cultured FLC-4 cells, which are derived from HCC, with iCDM-34 treatment resulted in increased expression of *CYP1A2* (Fig. 7A). These results suggest that iCDM-34 acts as an AhR agonist.

**Fig. 8:**
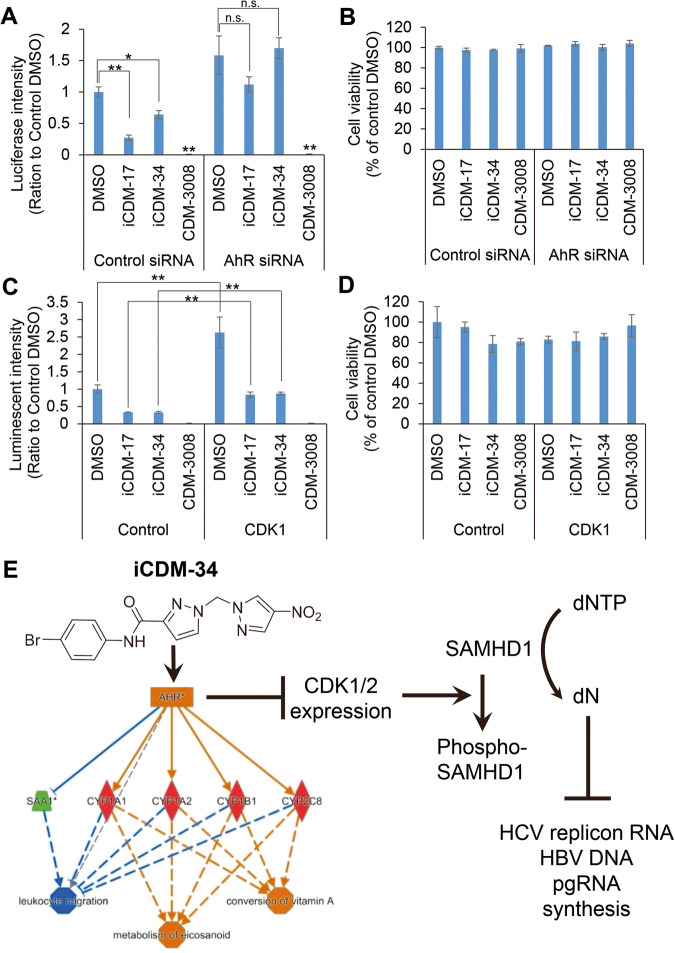
iCDM-34 induces AhR signaling based on AhR knockdown and CDK1 overexpression experiments. **A**, **B** HCV replicon cells were transfected with control and AhR siRNA. After 2 days of transfection, the cells were treated with 30 μM iCDM-17, iCDM-34, and CDM-3008 for 2 days. HCV replicon RNA levels were measured using a luciferase assay (**A**). Cell viability was measured using an XTT assay (**B**). **C**, **D** HCV replicon cells were transfected with control and CDK1 expression vectors. After 2 days of transfection, the cells were treated with 30 μM iCDM-17, iCDM-34, and CDM-3008 for 2 days. HCV replicon RNA levels were measured using a luciferase assay (**C**). Cell viability was measured using an XTT assay (**D**). Error bars indicate the SD (*n* = 3). The data are presented as the means ± SD. *p < 0.05 and **p < 0.01 based on two-tailed Student’s *t* test. n.s. indicates not significant. **E** iCDM-34 induces AhR activation and anti-HBV and anti-HCV activities via SAMHD1. iCDM-34 activates AhR which suppresses CDK1/2 expression, and SAMHD1 promotes the transition of dNTP to dN. Decreased dNTP levels inhibit HCV replicon RNA, HBV DNA, and pgRNA synthesis.

Kueck et al. reported that AhR activation inhibits HIV-1 and HSV-1 replication by repressing CDK1/2, thereby reducing SAMHD1 phosphorylation and cellular dNTP levels [12]. To analyze the function of iCDM-34 in suppressing HBV/HCV replication, phospho-SAMHD1 levels in HepG2-NTCP-C4 cells were analyzed by Western blotting after 1 day of treatment with iCDM-17 and iCDM-34, and were observed to be lower than pre-treatment levels (Fig. 7B, Supplemental Fig. 2). Phosphorylation of SAMHD1 is mediated by CDK1/2, which are suppressed by AhR activation (Fig. 8E) [12]. Thus, CDK1/2 expression levels were analyzed by qPCR after 3, 8, and 24 h of treatment with iCDM-17 and iCDM-34 (Fig. 7C–H). Suppression of CDK1 levels began after 3 h of iCDM-34 treatment, and significant suppression occurred after 8 and 24 h (Fig. 7C–E). CDK2 expression levels were significantly suppressed after 3 and 8 h of treatment with iCDM-34, and continued to be suppressed after 24 h (Fig. 7F–H). Thus, iCDM-34 appears to exert its anti-HBV and anti-HCV activities through SAMHD1.

To confirm the mechanism underlying the effects of iCDM-34, we performed AhR knockdown and CDK1 overexpression experiments using HCV replicon cells. iCDM-34 demonstrated significant anti-HCV activity with control siRNA; however, this activity was not present under treatment with AhR siRNA (Fig. 8A). CDM-3008 exhibited anti-HCV activity with or without AhR siRNA treatment; this may be because CDM-3008 is an IFNAR2 agonist and its anti-HCV activity is independent of AhR signaling (Fig. 8A). In this knockdown experiment, the knockdown efficiency of AhR was approximately 70%, and significant cytotoxicity was not observed when compared to treatment with control DMSO (Fig. 8B). HCV replicon RNA was increased in DMSO-, iCDM-17-, and iCDM-34-treated CDK1-overexpressing cells when compared to control vector-transfected cells (Fig. 8C, D). Taken together, these results suggest that iCDM-34 activates AhR and suppresses CDK1/2 mRNA expression, thereby increasing the levels of SAMHD1 and dN. These in turn suppress newly synthesized HCV replicon RNA, HBV DNA, and pgRNA (Fig. 8E).

## Discussion

We first attempted to identify an IFNAR2 agonist using the pocket structure of the IFN-α-binding site within IFNAR2-ECD, but the results of microarray gene expression analysis revealed that iCDM-34, which was identified by in silico screening, acts as an activator for AhR without activating the IFN signal. iCDM-34 could suppress HBsAg, and HBeAg; thus, it may have the potential to be a functional cure.

### Limitations and potential sources of bias and confounding factors in the in silico screening

We used in silico screening to identify small molecules that bind to the extracellular domain (ECD) of IFNAR2 to activate IFN signaling, but could not identify any compounds which activated IFN signaling. The 3D structure of the ECD of IFNAR2 complexed with IFN-α2 and H_2_O (PDB ID: 3S9D), but IFN-α2 and H_2_O were removed from the structure used for in silico screening. The 3D structure of the ECD of IFNAR2 is assumed to change depending on the presence or absence of IFN-α2 and H_2_O, and the 3D structure used for screening is a potential confounding factor. In addition, the IFN-α2 binding pocket in the ECD of IFNAR2 was used as the binding site for small molecules in this screening, but the CDM-3008-binding site in the ECD of IFNAR2 has not been determined [10]. Thus, the binding pocket used for in silico screening is a potential source of bias. In addition, due to limitations of the computer processing power used for screening, H_2_O could not be added to the simulation; this limited our ability to accurately predict docking. We believe that the use of a supercomputer with improved performance will address some of the limitations of this study.

### AhR agonist for induction of antiviral activity

AhR agonists bind to AhR and enter the nucleus to induce the expression of *CYP1A1*, *CYP1A2*, and *CYP1B1*. Kueck *et al*. showed that SAMHD1 metabolizes dNTP to dN, which is suppressed by CDK1-dependent phosphorylation of SAMHD1. In addition, CDK1 expression is suppressed by AhR agonist, and HIV-1 and HSV-2 replication was inhibited [12]. Hu et al. reported that CDK2 phosphorylates SAMHD1 regulating HBV replication [13]. Thus, AhR agonists could mediate the metabolism of dNTP to dN and regulate viral replication [12]. In HBV replication, *SAMHD1* knockdown increases extracellular and intracellular HBV DNA contents in Hep2.2.15 cells and HBV-infected HepG2-NTCP cells, whereas overexpression of *SAMHD1* reduces extracellular and intracellular HBV DNA contents [14, 15]. We showed that iCDM-34 reduced phospho-SAMHD1 level. This suggests that iCDM-34 also works as an activator for AhR and suppresses HBV replication through SAMHD1-dependent dNTP reduction.

It has been reported that the AhR agonist 2,3,7,8-tetrachlorodibenzo-p-dioxin enhances the production of HCV. In contrast, the AhR antagonist CH-223191 inhibits the expression of *CYP1A1* and decrease lipid droplet formation, thereby suppressing HCV replication [16]. However, iCDM-34 suppressed HCV and HBV replication (Fig. 1), suggesting that iCDM-34 suppress HCV and HBV mainly through the suppression of phospho-SAMHD1. iCDM-34 appeared to be an AhR agonist based on Ingenuity Pathway Analysis (IPA) and *CYP1A2* expression in the 3D cultured FLC-4 cells (Figs. 5, 7). AhR agonists can inhibit the replication of not only HIV-1 but also herpes simplex virus type 1 [12]. However, it has been reported that some AhR agonists can promote HCV replication [16], and the results might differ depending on the mechanism of action, culture cell type, and chemical structure of the AhR agonist.

Phosphoprotein proteomic analysis showed that phosphorylated ADAR was highly suppressed with iCDM-34 treatment (Fig. 4C lower panel). Nakano et al. reported that AhR mRNA edited by ADAR makes microRNA recognition sequence, and then AhR expression is suppressed [17]. These results suggest that iCDM-34 suppresses ADAR activation, and then microRNA-dependent degradation of AhR mRNA is also suppressed. As a result, AhR downstream pathway appears to be activated with iCDM-34 treatment.

### Anti-HBV activity of iCDM-34 in cultured cells

Nucleotide analogs and IFN drugs have been employed for HBV treatment. Nucleotide analogs can inhibit HBV replication but not cccDNA, HBsAg, or HBeAg, and may cause viral reactivation. On the other hand, IFN-α and IFN-γ treatment have been reported to degrade cccDNA through the adenosine deaminase activity of APOBEC3 [18, 19]. However, IFN agents are effective in only about 30% of patients and side effects are reported [3]. CDM-3008, an IFNAR2 agonist, has been reported to activate the JAK/STAT pathway and degrade cccDNA through the IFN signaling pathway [7]. Some capsid formation inhibitors, such as ABI-H0731 and JNJ-6379, inhibit the synthesis of HBV DNA from *pgRNA* by inhibiting capsid formation and suppress the de novo synthesis of cccDNA [20, 21]. In addition, the inactivation of cccDNA by genome editing using CRISPR/Cas9 has been actively examined [22], and a complete cure by the degradation of cccDNA is being investigated.

In this study, we showed that iCDM-34 inhibited *pgRNA*, HBsAg, and HBeAg, which are markers of cccDNA (Figs. 2, 5). Microarray gene expression analysis suggests that iCDM-34 is an activator for AhR and suppresses HBV DNA and pgRNA by a mechanism completely different from that of IFN-α (Fig. 5). iCDM-34 (KUSC-5001) can also suppress the promoter of hypoxia inducible factor (HIF) [23]. Wing et al. reported that HIFs activate HBV replication through the basal core promoter [24]. Thus, iCDM-34 could suppress *pgRNA*, HBeAg, and HBV DNA through the inhibition of the HIF promoter.

### Anti-HBV activity of iCDM-34 in human hepatocytes chimeric mice

In experiments using human hepatocyte-chimeric mice, iCDM-34 did not show anti-HBV activity as a single agent, but suppressed HBV DNA when combined with ETV (Fig. 3C, D). The stability of iCDM-34 was examined using liver microsomal fractions, and its half-life was found to be 505.8 min in humans and 33.1 min in mice, indicating high stability in humans. Metabolic experiments using human hepatocyte-chimeric mice showed that the half-life of iCDM-34 was 23 min in blood (Fig. 3A, B). Owing to the high metabolic rate, iCDM-34 cannot be used as a single drug; however, it exhibited additively inhibitory effects with ETV. We will continue the structure–activity relationship study of iCDM-34 to identify compounds that have higher activity and metabolic stability.

## Conclusion

iCDM-34 with a new pharmacophore clearly showed inhibition of viral replication, and is a promising antiviral agent. However, further analysis of the virus suppression mechanism is needed to use iCDM-34 as an HBV therapeutic agent. In addition, we will confirm its antiviral activity against coronaviruses and flaviviruses in the future, and develop iCDM-34 for use as a therapeutic agent in the event of a possible pandemic.

## Materials and methods

### Materials

iCDM-17 (STK781802) and iCDM-34 (STK403994) were purchased from Vitas-M Laboratory (Hong Kong). PegIFN-α2a was purchased from Chugai Pharmaceutical (Tokyo, Japan).

### Molecular docking simulations

Prior to molecular docking calculations, we constructed the IFNAR2 model structure using the X-ray crystallographic structure of IFNAR2 (PDB ID: 3S9D) [25]. IFN-α2 and water molecules were removed, and the protein structure was prepared using Schrödinger Protein Preparation Wizard of maestro module (Schrödinger, New York, NY, USA) [26]. Docking center was set to the loop that included Tyr39 and Tyr43. We used a compound library for docking simulations provided by Namiki Shoji (Tokyo, Japan). The compound library was filtered using Lipinski’s rule of five, resulting in exclusion of compounds with reactive groups and selection of approximately 300,000 compounds at random. Alternative protonation states of each compound as well as chiral forms were generated for the 7 ± 2 pH range using the LigPrep module and ionization penalties were calculated using the Epik panel (Schrödinger, NY, USA) at pH 7 [27]. The docking simulations were performed using GOLD with GoldScore (The Cambridge Crystallographic Data Center, Cambridge, UK) [28], Glide (SP mode) with GlideScore (Schrödinger, NY, USA) [29], and Molecular Operating Environment (MOE) Docking score (S score) (Chemical Computing Group, Montreal, Canada) [30].

### Cell culture

HCV replicon cells [31] were plated on clear and white 96-well plates (TPP, 92096 and Greiner Bio-one, 655083) in DMEM, high glucose, GlutaMAX Supplement (Thermo Fisher Scientific, Waltham, MA, USA) containing 10 % fetal bovine serum, penicillin, and streptomycin. After 1 day, the cells were treated with 0.05–30 µM of iCDM-17 and iCDM-34 for 3 days. The quantity of HCV replicons was measured by a luciferase assay using Steady-Glo Luciferase Assay System (Promega, Madison, WI, USA). The cell viability was measured by XTT assay using Cell proliferation kit II (Roche, Mannheim, Germany) according to the established protocol [7].

For knockdown of AhR in the HCV replicon cells, 2 ×10^4^ cells of HCV replicon cells were mixed with AhR siRNA (siGENOME Human AHR siRNA – Smart pool, Horizon Discovery, Cambridgeshire, United Kingdom) and X-tremeGene 360 transfection reagent (Roche) and plated on 96 well plates. After 2 days of knockdown, the cells were treated with 30 μM iCDM-17, iCDM-34, and CDM-3008 for 2 days. The quantity of HCV replicons and cell viability were measured. The cellular RNA was purified using Agencourt RNAdvance (Beckman Coulter, San Jose, CA) and reverse-transcribed using PrimeScript RT master mix (Takara Bio, Kusatsu, Japan) to measure amount of AhR and β-actin mRNAs by quantitative PCR.

For overexpression of CDK1, CDK1 coding region was inserted into pCAG-Neo-PA tag-C expression vector (Fuji Film, Tokyo, Japan) at EcoRI and BamHI site. HCV replicon cells were transfected with pCAG-CDK1-PA expression vector and control vector (pCAG-Neo-PA) using X-tremeGene 360 (Roche). After 1 day of transfection, the cells were treated with 30 μM iCDM-17, iCDM-34, and CDM-3008 for 3 days. The quantity of HCV replicons and cell viability were measured.

HepG2-NTCP-C4 cells were plated on a 96-well plate in DMEM/F12 and GlutaMAX Supplement (Thermo Fisher Scientific) containing 10% fetal bovine serum, 10 mM HEPES, 5 μg/ml insulin, 50 mM hydrocortisone, 200 U/ml penicillin, and 200 μg/ml streptomycin. After 1 day, the cells were treated with 30 μM iCDM-17 and iCDM-34 for 3, 8, 24 h. Total RNA was purified form the cells using Agencourt RNA advance (Beckman Courter). cDNA was synthesized using PrimeScript RT master mix (Takara Bio). CDK1, CDK2, β-actin mRNA amount was quantified by qPCR.

PXB cells (primary cultured human hepatocytes) were purchased from PhoenixBio (Higashi-Hiroshima, Japan) and cultured on collagen type I-coated 96- or 24-well plates in hepatocyte clonal growth medium at 37 °C and 5 % CO_2_ as previously described [32]. RealTime-Glo MT Cell Viability Assay (Promega) and XTT assay were performed according to a previously established protocol [7].

### qPCR analysis of ISGs and AhR signaling molecules

mRNA expression levels were measured by qPCR using specific primers or sets of specific primers and probe. RNA was reverse transcribed using PrimeScript RT Master Mix (Takara Bio, Shiga, Japan) to synthesize complementary DNA (cDNA). *OAS1*,glyceraldehyde 3-phosphate dehydrogenase (*GAPDH*), β-actin mRNA expression levels were measured using specific primers and TB Green Premix Ex Taq II (Takara Bio). *AhR* (Hs00169233_m), *CDK1* (Hs00938777_m), *CDK2* (Hs01548894_m) mRNA expression levels were measured using specific probe and primer sets (Thermo Fisher Scientific, Waltham, MA, USA) and Probe qPCR mix (Takara Bio) in a CFX96 Touch Real-Time PCR Detection System (Bio-Rad Laboratories, Hercules, CA, USA). The specific primers used in this study were *OAS1* (forward primer: 5′-TCCGTGAAGTTTGAGGTCCA-3′, reverse primer: 5′-ATCAAAGGCAGGCAGCACAT-3′), reverse primer: 5′-CGAACACCTGAATCAAGGAGTTA-3′, and *GAPDH* (forward primer: 5′-CAATGACCCCTTCATTGACC-3′, reverse primer: 5′-GACAAGCTTCCCGTTCTCAG-3′). *β-actin* (forward primer: forward primer: 5′-AGAAAATCTGGCACCACACC-3′ and reverse primer: 5′-AAGGTCTCAAACATGATCTGGG-3′). Expression levels were determined using the 2^–ΔΔCt^ method and were normalized to endogenous *β-actin* mRNA expression levels.

### Assay of anti-HBV activity using PXB cells

PXB cells were plated on 96-well plate and infected with three genome equivalents per cell of HBV C_AT [33], and the medium was changed every 3 or 4 days. After 14–28 days of infection, the cells were then treated with a final concentration of 0.0003–30 µM of iCDM-17, iCDM-34 and CDM-3008 and 10 ng/mL pegIFN-α2a for 7 days. After treatment with these compounds, cellular DNA, medium DNA, and total RNA were automatically purified as per our previously established protocol [7]. RNA purification of total RNA from cultured cells in the 24-well plate was performed using the RNeasy Mini Kit (Qiagen, Germantown, MD, USA) according to the manufacturer’s instructions.

### Quantitative PCR (qPCR) analysis of HBV DNA

HBV DNA copy numbers were determined using TaqMan Gene Expression Master Mix (Thermo Fisher Scientific) or Probe qPCR Mix (Takara Bio), specific primers, and probes in a qPCR system (Light Cycler 96 System, Roche, Basel, Switzerland or CFX96 Touch Real-Time PCR Detection System, Bio-Rad Laboratories, Hercules, CA, USA). The specific primers and probe used for detection of HBV DNA were as follows: forward primer: 5′-ACTCACCAACCTCCTGTCCT-3′, reverse primer: 5′-GACAAACGGGCAACATACCT-3′, and probe: 5′-[FAM] TATCGCTGGATGTGTCTGCGGCGT[TAM]-3′.

### Phosphopeptides Enrichment and iTRAQ Labeling

HCV replicon cells were plated on a 10-cm plate in DMEM, high glucose, GlutaMAX Supplement (Thermo Fisher Scientific, Waltham, MA) containing 10% fetal bovine serum, penicillin, and streptomycin. After 1 day, the cells were treated with 30 µM of 2% DMSO, CDM-3008, iCDM-17, and iCDM-34 for 24 hr. Then, the cells were washed with phosphatase inhibitors in PBS (1 mM Na_3_VO_4_, 10 mM NAF, 10 mM Na_4_P_2_O_7,_ Na_2_MoO_4_, *β*-*glycerophosphate*). The cells were scraped and harvested by centrifugation and lysed in 9.8 M urea with a protease inhibitor cocktail and a phosphatase inhibitors cocktail. The lysed cell was sonicated, and the clear lysate was collected by centrifugation. The protein concentration was measured by BCA protein assay. For each sample, ovalbumin was added as an internal standard. Then, the total lysate protein with the standard was treated with 2 mM TCEP for 30 min at 37 °C and then alkylated in the presence of 55 mM iodoacetamide for 30 min with light protection. The mixtures were trypsinized at an enzyme-to-substrate ratio of 1:200 overnight at 37 °C. The digested mixtures were acidified by adding 0.25% TFA and subsequently desalted using C18 Sep-Pak columns (50 mg sorbent weight, Waters). Enrichment of phosphopeptides was performed as described previously [34]. Briefly, the peptides from each sample were reconstituted in immobilized metal affinity chromatography (IMAC) incubating buffer (60% ACN containing 0.1% TFA. IMAC beads were prepared as follows; Nickel ions in Ni-NTA agarose beads (Qiagen, Valencia, CA) were removed by incubation with 50 mM EDTA, washed with H_2_O and subsequently replaced by Fe^3+^ ions. The beads were then washed with 0.1% Acidic acid followed by IMAC incubating buffer. IMAC beads were mixed with peptides and incubated for 2 h at room temperature with rotation. Thereafter, the beads were washed with IMAC incubating buffer followed by washing buffer containing 2% ACN plus 0.1% TFA. Phosphopeptides were eluted stepwise by 100 mM phosphoric acid pH 2.7 followed by 0.5% NH_4_OH. Two eluted fractions were combined and neutralized by TFA. Phosphopeptides were desalted via C18 Sep-Pak columns (50 mg sorbent weight, Waters). Samples were lyophilized to dryness. All samples were then chemically labeled with iTRAQ reagent (AB Sciex, Framingham, MA). The iTRAQ labeled phosphopeptides were then combined and desalted by C18 StageTips. Samples were lyophilized and fractionated into 7 fractions by using SDB-XC StageTips. Each fraction was dried under vacuum and dissolved in the measurement buffer (3% Acetonitrile (ACN) and 0.1% Formic acid (FA)).

### Data processing

All raw files acquired in this study were collectively processed with Proteome Discoverer 2.5 (Thermo Fisher Scientific, Waltham, MA). Database searching for peptide and protein identification was performed using Mascot 2.7 (Matrix Science, London, UK) against UniProt human database (released on January 2021). The precursor mass tolerance was set to 20 ppm, and the fragment mass tolerance was set to 0.02 Da. Carbamidomethylation of cysteine was set as a fixed modification and oxidation of methionine, deamidation of asparagine and glutamine, phosphorylation on serine, threonine and tyrosine were allowed as variable modifications. The confident phosphorylation site localization using IMP-ptmRS node was used [35, 36]. The site probability threshold was set to 75 as a default workflow of Proteome Discoverer 2.5. Moreover, iTRAQ labelings of N-terminus and lysine were set as fixed modifications and iTRAQ labeling of tyrosine was set as a variable modification. Up to two missed cleavages were allowed. A false discovery rate (FDR) of a maximum of 1% was selected for peptide identifications. Normalization between samples was performed using an internal standard; ovalbumin.

### Analysis of biological functions and signaling pathways

A list of phosphopeptides was exported from Proteome Discoverer 2.5 (Thermo Fisher Scientific). As responses to the treatment of iCDM-34, we selected phosphopeptides with the fold changes of the iCDM-34 treatment/DMSO below 0.67 or over 1.5 and also without the fold changes of the CDM-3008 or iCDM-17 treatment/DMSO below 0.67 or over 1.5. The alterations of phosphoproteins were analyzed by Ingenuity Pathway Analysis (IPA) (Qiagen). The top fifteen biological functions altered by the iCDM-34 treatment were listed in comparison with other treatments. The illustration was drawn by Prism version 7.0 (GraphPad Software Inc., San Diego, CA).

### Measurement of HBV antigens

HBsAg and HBeAg levels in the cell culture medium were measured using Enzygnost HBsAg 6.0 and Enzygnost HBe monoclonal (Siemens, Munich, Germany), respectively, according to the manufacturer’s instructions.

### Microarray analysis

PXB cells cultured in 24-well plates were infected with three genome equivalents per cell of HBV C_AT. After 24 days of infection, the cells were treated with 0.3 and 30 µM iCDM-34 and 1,000 IU/mL IFN-α (Sumitomo Dainippon Pharma, Osaka, Japan) for 7 days (*n* = 3). Total RNA was extracted from the treated cells and subjected to microarray analysis [7]. The raw and normalized data were uploaded to NCBI Gene Expression Omnibus (GSE180646).

### Knowledge-based pathway analysis

To explore the biological interpretation of the transcriptome data, the canonical pathways and upstream causal networks were identified using knowledge-based functional analysis software from Ingenuity Pathways Analysis (IPA; Ingenuity Systems, Redwood City, CA, USA). In the IPA analysis, the fold-change was set a ratio (case/control), and the gene was considered upregulated if the fold-change was between 1 and +infinity, and the value (x) between 0 and 1 was converted as “-1/x”. The gene was considered downregulated if the fold-change was between -infinity to -1. A statistical measure of the IPA analysis is the activation z-score, which can be used to identify likely regulating molecules based on a statistically significant pattern match of up- and downregulation, and to predict the activation state (either activated or inhibited) of a putative regulator [37]. An absolute value of z-score more than 2 was considered significant.

### Western blot analysis

HCV replicon cells were treated with 30 μM CDM-3008, iCDM-17, and iCDM-34 for 1 day. The cells were lyzed with 1 x SDS sample buffer containing PhosStop, Complete EDTA free, and 1 mM EDTA. After sonication for 30 s, the lysates were centrifuged at 12,000*g* for 10 min at 4 °C, and then the supernatants were collected. After reducing the 20 μg of supernatants with 50 mM DTT at 98 °C for 2 min, the samples were separated by SDS-PAGE. STAT1, STAT2, STAT3, pSTAT1, pSTAT2, and pSTAT3 were detected by Western blotting using anti-STAT1 (9175, 1/1000), STAT2 (72604, 1/1000), STAT3 (12640, 1/1000), pSTAT1 (Y701) (7649, 1/1000), pSTAT2 (Y690) (88410, 1/1000), and pSTAT3 (Y705) (9145, 1/1000) antibodies (Cell signaling technology, Massachusetts, USA).

HepG2-NTCP-C4 cells were treated with 30 μM iCDM-17 and iCDM-34 for 1 day. The cells were lyzed with 20 mM TrisHCl pH 7.5 containing 100 mM NaCl, 10 mM EDTA, 1% TritonX-100, 1% Sodium deoxycholate, PhosSTOP (Roche), Complete EDTA free. The lysates were sonicated 30 s and mixed for 1 h with rotation at 4 °C. The lysates were centrifuged at 15,000 rpm for 10 min at 4 °C. The supernatants were analyzed by Western blotting using anti-phospho-SAMHD1 (Thr592) (Cell signaling technology, 89930, 1/1000), anti-SAMHD1 (Cell signaling technology, 49158, 1/1000), and β−actin (Santa Cruz, 1/1000).

### 3D culture of FLC-4 cells and CYP1A2 quantitative PCR (qPCR)

The human hepatocellular carcinoma cell line FLC-4 was cultured on micro-space cell culture plates (Elplasia SQ-200, -100, -24, Kuraray, Tokyo, Japan) maintained at 37 °C and 5 % CO_2_ according to the manufacturer’s protocol. FLC-4 cells suspended in DMEM/Ham’s F12 medium supplemented with 10% heat-inactivated fetal bovine serum, 100 U/mL penicillin G, and 100 μg/mL streptomycin were seeded at a density of 8.0 × 10^5^ cells/well and cultured for 3 days. The medium was replenished every 72 h, completely removed, and replaced with serum-free medium containing 1–10 µM iCDM-34 or vehicle (DMSO). Total RNA was isolated from cells and reverse transcribed complementary DNA was subjected to quantitative qPCR. mRNA expression was determined using TaqMan Gene Expression Assays (Hs00167927_m1 for CYP1A2, Hs00604506_m1 for CYP3A4, for Hs00426397_m1 for CYP2C9; Thermo Fisher Scientific, Waltham, MA, USA). Expression levels were determined using the 2^–ΔΔCt^ method and were normalized to endogenous *GAPDH* mRNA expression levels.

### Multivariate and statistical analyses

Unsupervised principal component analysis was performed using MetaboAnalyst version 5.0 (https://www.metaboanalyst.ca/). Student’s *t*-test was performed to compare means using Microsoft Excel 2013/2016 (Microsoft, Redmond, WA, USA). Mann-Whitney test was performed using Graph Pad Prism 9 (GraphPad Software, San Diego, CA).

### Liver microsomal stability studies

Liver microsome stability studies (human, mouse, and rat) were performed at Sumika Chemical Analysis Service (Osaka, Japan) by following an established procedure [38]. A mixture of 1 μM of substrate, 0.5 mg protein/mL of microsomes, and 3.5 μM β-NADPH was incubated for 30 or 60 min and % remaining substrate was quantitatively measured by LC-MS/MS.

### Assay of iCDM-34 anti-HBV activity in human hepatocyte-chimeric mice

PXB-mice (human liver chimeric cDNA-uPA^wild/+^/SCID male mice) were generated by PhoenixBio. HBV genotype C was then inoculated into the mice via i.v. [33]. After attaining 1.2 × 10^8^–3.5 × 10^8^ copies/mL of the serum HBV DNA levels, vehicle (10% 2-hydroxypropyl-beta-cyclodextrin and 10% polyethylene glycol 300) or 1 mg/kg iCDM-34 was i.v. injected with or without administration of 0.03 mg/kg ETV orally once a day for 21 days (*n* = 4). DNA was purified from the liver and its concentration was measured using NanoDrop Spectrophotometer (Thermo Fisher Scientific), followed by measurement of HBV DNA levels by qPCR according to a previously established method [7]. The experimental procedures using the PXB-mice was approved by the Animal Ethics Committee of PhoenixBio.

### Supplementary information

Supplemental Legends and Figures

Original Data File

## Data Availability

The data analyzed during this study are included in this published article and the supplemental data files. The raw and normalized data were uploaded to NCBI Gene Expression Omnibus (GSE180646).

## References

[1] World Health Organization. Hepatitis B fact sheets. World Health Organization; 2022.

[2] Ohishi W, Chayama K (2012). Treatment of chronic hepatitis B with nucleos(t)ide analogues. Hepatol Res.

[3] Perrillo R (2009). Benefits and risks of interferon therapy for hepatitis B. Hepatology.

[4] Lee HW, Lee JS, Ahn SH. Hepatitis B Virus cure: targets and future therapies. Int J Mol Sci. 2020;22:213. 10.3390/ijms22010213 PMC7795643 33379331

[5] Stadler D, Kächele M, Jones AN, Hess J, Urban C, Schneider J (2021). Interferon-induced degradation of the persistent hepatitis B virus cccDNA form depends on ISG20. EMBO Rep.

[6] Amin OE, Colbeck EJ, Daffis S, Khan S, Ramakrishnan D, Pattabiraman D, et al. Therapeutic potential of TLR8 agonist GS-9688 (selgantolimod) in chronic hepatitis B: re-modelling of antiviral and regulatory mediators. Hepatology. 2020;75:54–71. 10.1002/hep.31695 PMC8436741 33368377

[7] Furutani Y, Toguchi M, Shiozaki-Sato Y, Qin XY, Ebisui E, Higuchi S (2019). An interferon-like small chemical compound CDM-3008 suppresses hepatitis B virus through induction of interferon-stimulated genes. PLoS ONE.

[8] Ito K, Okumura A, Takeuchi JS, Watashi K, Inoue R, Yamauchi T, et al. Dual agonist of farnesoid X receptor and Takeda G protein-coupled receptor 5 inhibits Hepatitis B virus infection in vitro and in vivo. Hepatology. 2021;74:83–98. 10.1002/hep.31712 33434356

[9] Niu C, Li L, Daffis S, Lucifora J, Bonnin M, Maadadi S (2018). Toll-like receptor 7 agonist GS-9620 induces prolonged inhibition of HBV via a type I interferon-dependent mechanism. J Hepatol.

[10] Konishi H, Okamoto K, Ohmori Y, Yoshino H, Ohmori H, Ashihara M (2012). An orally available, small-molecule interferon inhibits viral replication. Sci Rep.

[11] Furutani Y, Toguchi M, Higuchi S, Yanaka K, Gailhouste L, Qin XY, et al. Establishment of a rapid detection system for ISG20-dependent SARS-CoV-2 subreplicon RNA degradation induced by interferon-α. Int J Mol Sci. 2021;22:11641. 10.3390/ijms222111641 PMC8583800 34769072

[12] Kueck T, Cassella E, Holler J, Kim B, Bieniasz PD. The aryl hydrocarbon receptor and interferon gamma generate antiviral states via transcriptional repression. Elife. 2018;7:e38867. 10.7554/eLife.38867 PMC6120754 30132758

[13] Hu J, Qiao M, Chen Y, Tang H, Zhang W, Tang D (2018). Cyclin E2-CDK2 mediates SAMHD1 phosphorylation to abrogate its restriction of HBV replication in hepatoma cells. FEBS Lett.

[14] Jeong GU, Park IH, Ahn K, Ahn BY (2016). Inhibition of hepatitis B virus replication by a dNTPase-dependent function of the host restriction factor SAMHD1. Virology.

[15] Sommer AF, Rivière L, Qu B, Schott K, Riess M, Ni Y (2016). Restrictive influence of SAMHD1 on Hepatitis B Virus life cycle. Sci Rep.

[16] Ohashi H, Nishioka K, Nakajima S, Kim S, Suzuki R, Aizaki H (2018). The aryl hydrocarbon receptor-cytochrome P450 1A1 pathway controls lipid accumulation and enhances the permissiveness for hepatitis C virus assembly. J Biol Chem.

[17] Nakano M, Fukami T, Gotoh S, Takamiya M, Aoki Y, Nakajima M (2016). RNA editing modulates human hepatic aryl hydrocarbon receptor expression by creating MicroRNA recognition sequence. J Biol Chem.

[18] Lucifora J, Xia Y, Reisinger F, Zhang K, Stadler D, Cheng X (2014). Specific and nonhepatotoxic degradation of nuclear hepatitis B virus cccDNA. Science.

[19] Xia Y, Stadler D, Lucifora J, Reisinger F, Webb D, Hösel M (2016). Interferon-γ and tumor necrosis factor-α produced by T cells reduce the HBV persistence Form, cccDNA, without cytolysis. Gastroenterology.

[20] Berke JM, Dehertogh P, Vergauwen K, Mostmans W, Vandyck K, Raboisson P, et al. Antiviral properties and mechanism of action studies of the Hepatitis B virus capsid assembly modulator JNJ-56136379. Antimicrob Agents Chemother. 2020;64. 10.1128/AAC.02439-19 PMC7179615 32094138

[21] Huang Q, Cai D, Yan R, Li L, Zong Y, Guo L, et al. Preclinical profile and characterization of the Hepatitis B virus core protein inhibitor ABI-H0731. Antimicrob Agents Chemother. 2020;64:e01463-20. 10.1128/AAC.01463-20 PMC7577125 32868329

[22] Dong C, Qu L, Wang H, Wei L, Dong Y, Xiong S (2015). Targeting hepatitis B virus cccDNA by CRISPR/Cas9 nuclease efficiently inhibits viral replication. Antivir Res.

[23] Sakai M, Takahashi N, Ikeda H, Furutani Y, Higuchi S, Suzuki T (2021). Design, synthesis, and target identification of new hypoxia-inducible factor 1 (HIF-1) inhibitors containing 1-alkyl-1H-pyrazole-3-carboxamide moiety. Bioorg Med Chem.

[24] Wing PAC, Liu PJ, Harris JM, Magri A, Michler T, Zhuang X, et al. Hypoxia inducible factors regulate hepatitis B virus replication by activating the basal core promoter. J Hepatol. 2021;75:64–73. 10.1016/j.jhep.2020.12.034 PMC8214165 33516779

[25] Thomas C, Moraga I, Levin D, Krutzik PO, Podoplelova Y, Trejo A (2011). Structural linkage between ligand discrimination and receptor activation by type I interferons. Cell.

[26] Schrödinger Release 2014-1. Maestro. New York, NY: Schrödinger, LLC; 2014.

[27] Schrödinger Release 2014-1. Epick. New York, NY: Schrödinger, LLC; 2014.

[28] Jones G, Willett P, Glen RC, Leach AR, Taylor R (1997). Development and validation of a genetic algorithm for flexible docking. J Mol Biol.

[29] Schrödinger Release 2014-1. Glide. New York, NY: Schrödinger, LLC; 2014.

[30] Molecular Operating Environment (MOE). 1010 Sherbooke St. West, Suite #910, Montreal, QC, Canada, H3A 2R7: 2014.01 Chemical Computing Group ULC; 2014.

[31] Watanabe T, Sudoh M, Miyagishi M, Akashi H, Arai M, Inoue K (2006). Intracellular-diced dsRNA has enhanced efficacy for silencing HCV RNA and overcomes variation in the viral genotype. Gene Ther.

[32] Ishida Y, Yamasaki C, Yanagi A, Yoshizane Y, Fujikawa K, Watashi K (2015). Novel robust in vitro hepatitis B virus infection model using fresh human hepatocytes isolated from humanized mice. Am J Pathol.

[33] Sugiyama M, Tanaka Y, Kato T, Orito E, Ito K, Acharya SK (2006). Influence of hepatitis B virus genotypes on the intra- and extracellular expression of viral DNA and antigens. Hepatology.

[34] Zhang Y, Wolf-Yadlin A, Ross PL, Pappin DJ, Rush J, Lauffenburger DA (2005). Time-resolved mass spectrometry of tyrosine phosphorylation sites in the epidermal growth factor receptor signaling network reveals dynamic modules. Mol Cell Proteom.

[35] Potel CM, Lemeer S, Heck AJR (2019). Phosphopeptide fragmentation and site localization by mass spectrometry: an update. Anal Chem.

[36] Taus T, Kocher T, Pichler P, Paschke C, Schmidt A, Henrich C (2011). Universal and confident phosphorylation site localization using phosphoRS. J Proteome Res.

[37] Qin XY, Hara M, Arner E, Kawaguchi Y, Inoue I, Tatsukawa H (2017). Transcriptome analysis uncovers a growth-promoting activity of Orosomucoid-1 on hepatocytes. EBioMedicine.

[38] Fonsi M, Orsale MV, Monteagudo E (2008). High-throughput microsomal stability assay for screening new chemical entities in drug discovery. J Biomol Screen.

