# Supplementary material for: A small molecule iCDM-34 identified by in silico screening suppresses HBV DNA through activation of aryl hydrocarbon receptor
Source: Cell Death Discov. 2023 Dec 22;9:467. doi: 10.1038/s41420-023-01755-w (PMC10746708; doi:10.1038/s41420-023-01755-w)
Supplement: Supplementary file 1 — Supplemental Legends and Figures [file 41420_2023_1755_MOESM1_ESM.pdf]

Supplemental Figure Legends

Supplemental Figure 1. HCV replicon cells were treated with 30 μM CDM-3008, iCDM-17, and iCDM-34 for 1 day. Expression levels of phospho-STAT1, STAT1, phospho-STAT2, STAT2, phospho-STAT3, and STAT3 were analyzed by Western blotting.

Supplemental Figure 2. HepG2-NTCP-C4 cells were treated with 30 μM iCDM-17 and iCDM-34 for 1 day. Expression levels of phospho-SAMHD1, SAMHD1, and β-actin were analyzed by Western blotting.

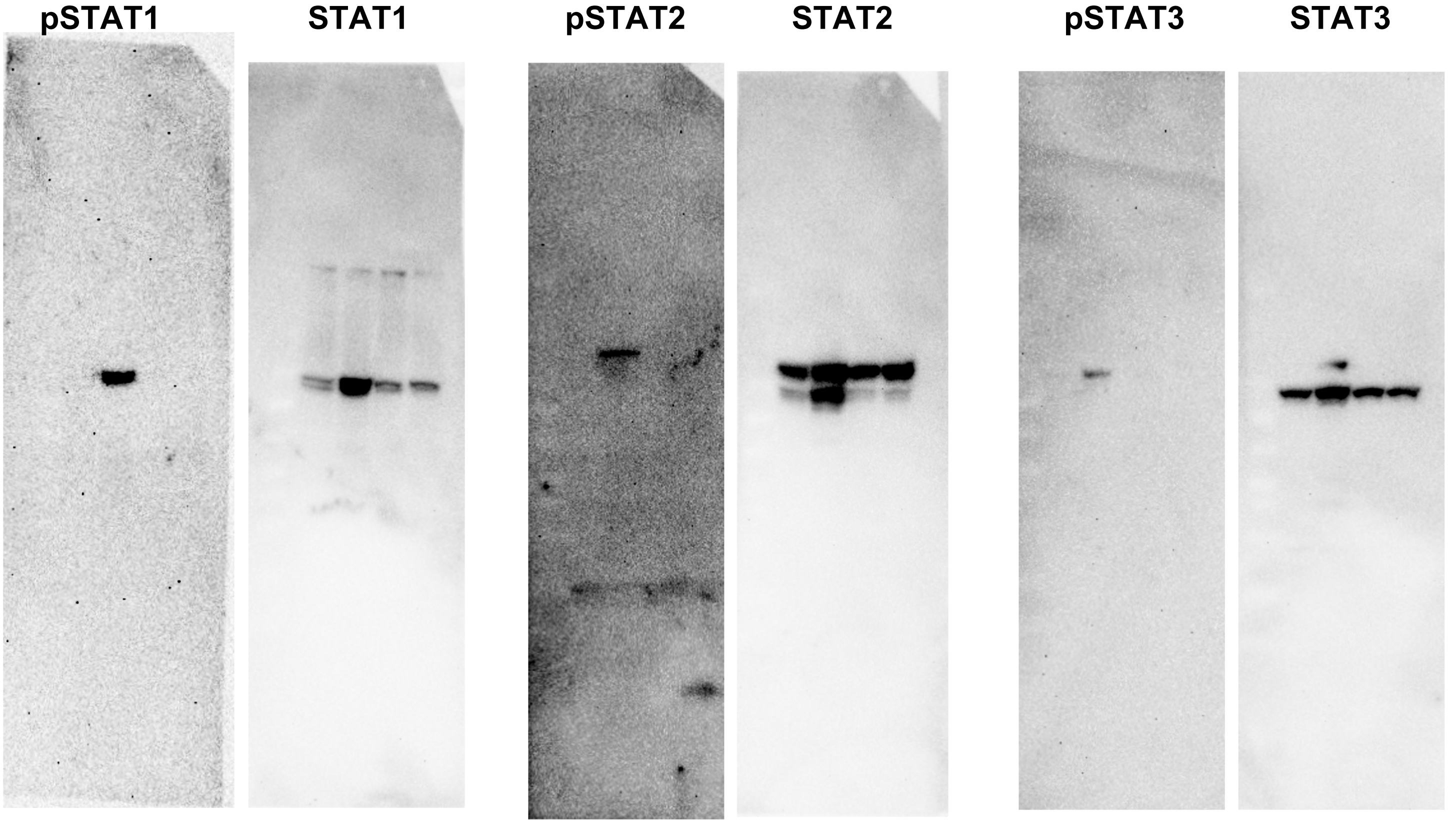

Supplemental Fig.1

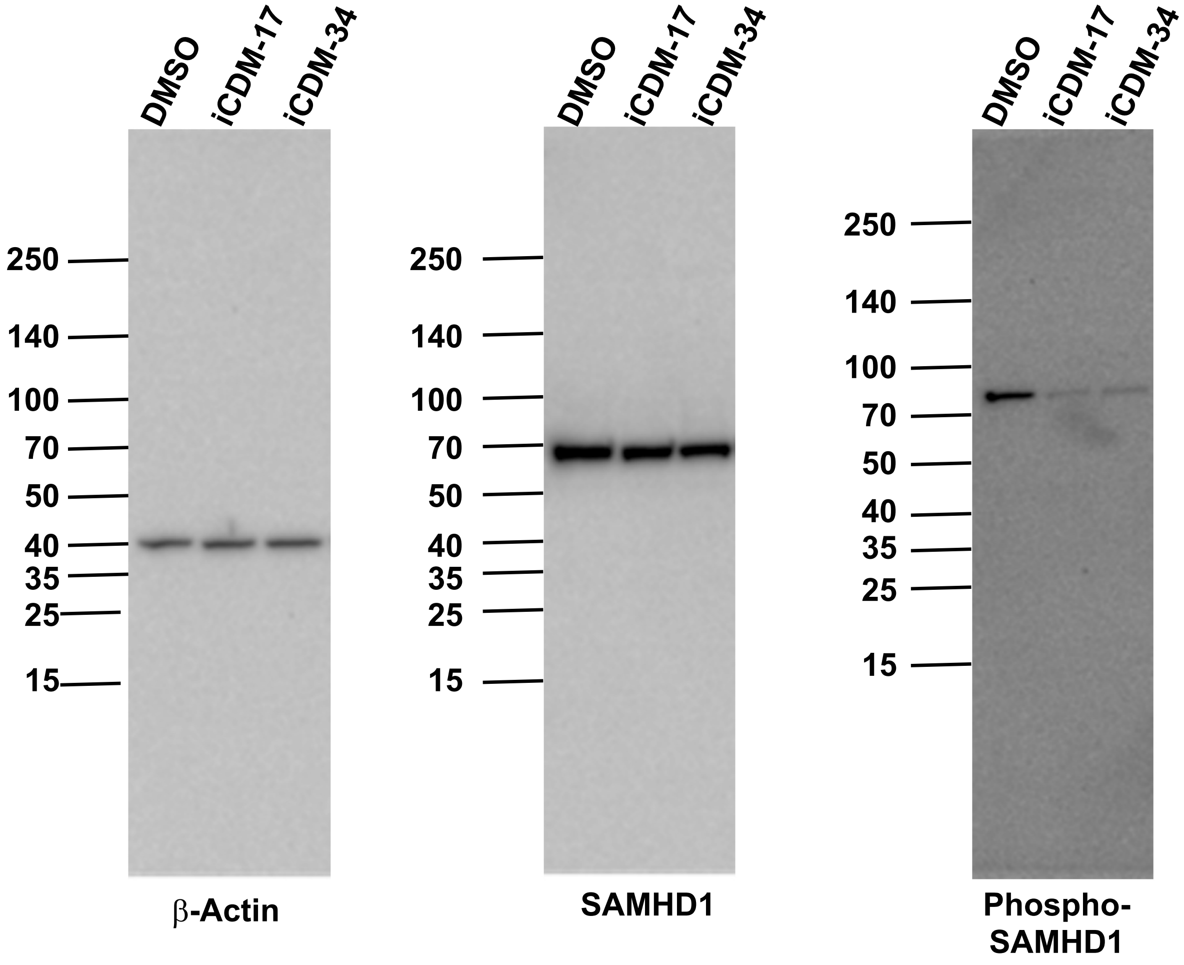

Supplemental Fig. 2
