# Supplementary figures and images for: A small molecule iCDM-34 identified by in silico screening suppresses HBV DNA through activation of aryl hydrocarbon receptor
Source: Cell Death Discov. 2023 Dec 22;9:467. doi: 10.1038/s41420-023-01755-w (PMC10746708; doi:10.1038/s41420-023-01755-w)

Original Data for Figure 4B

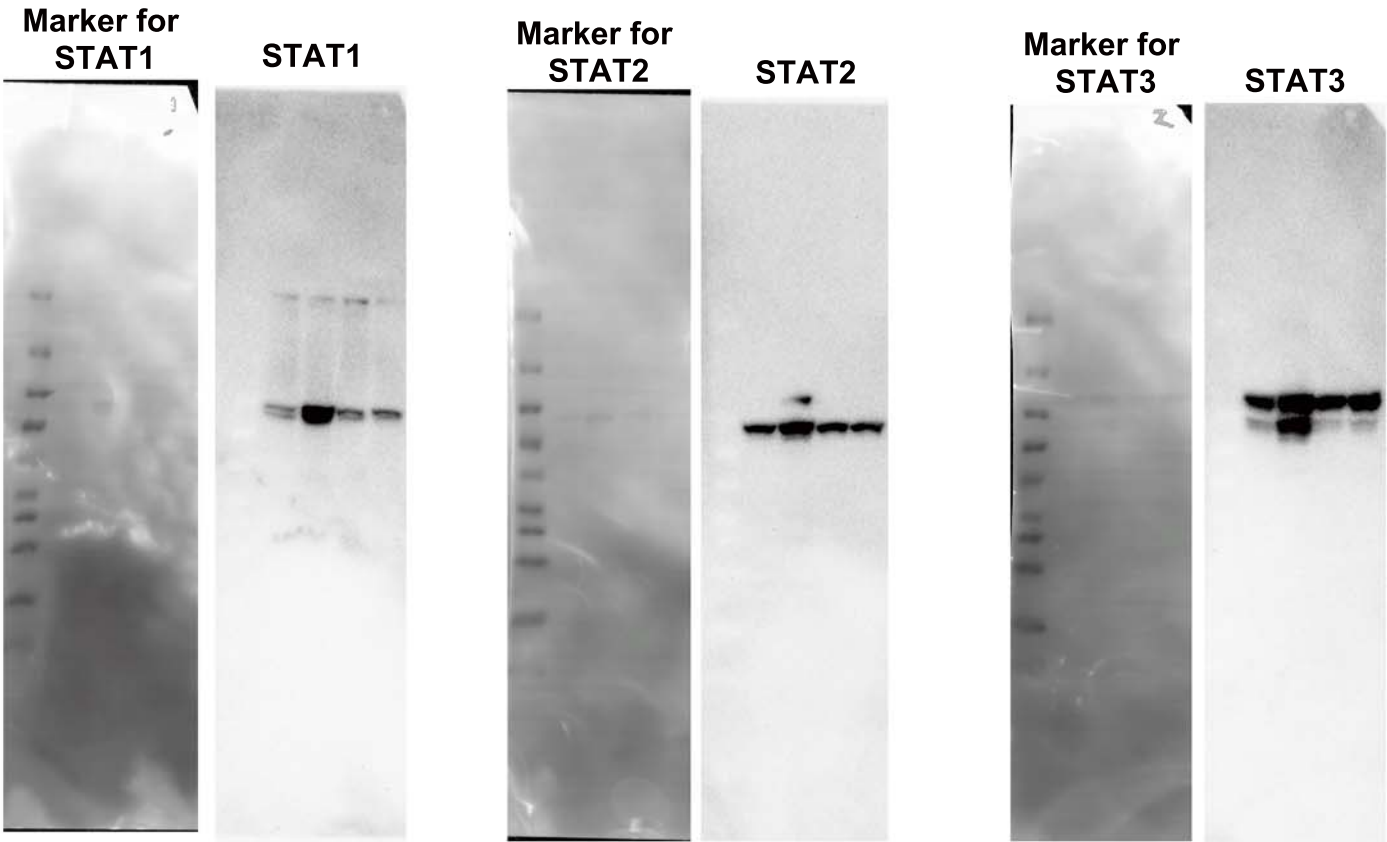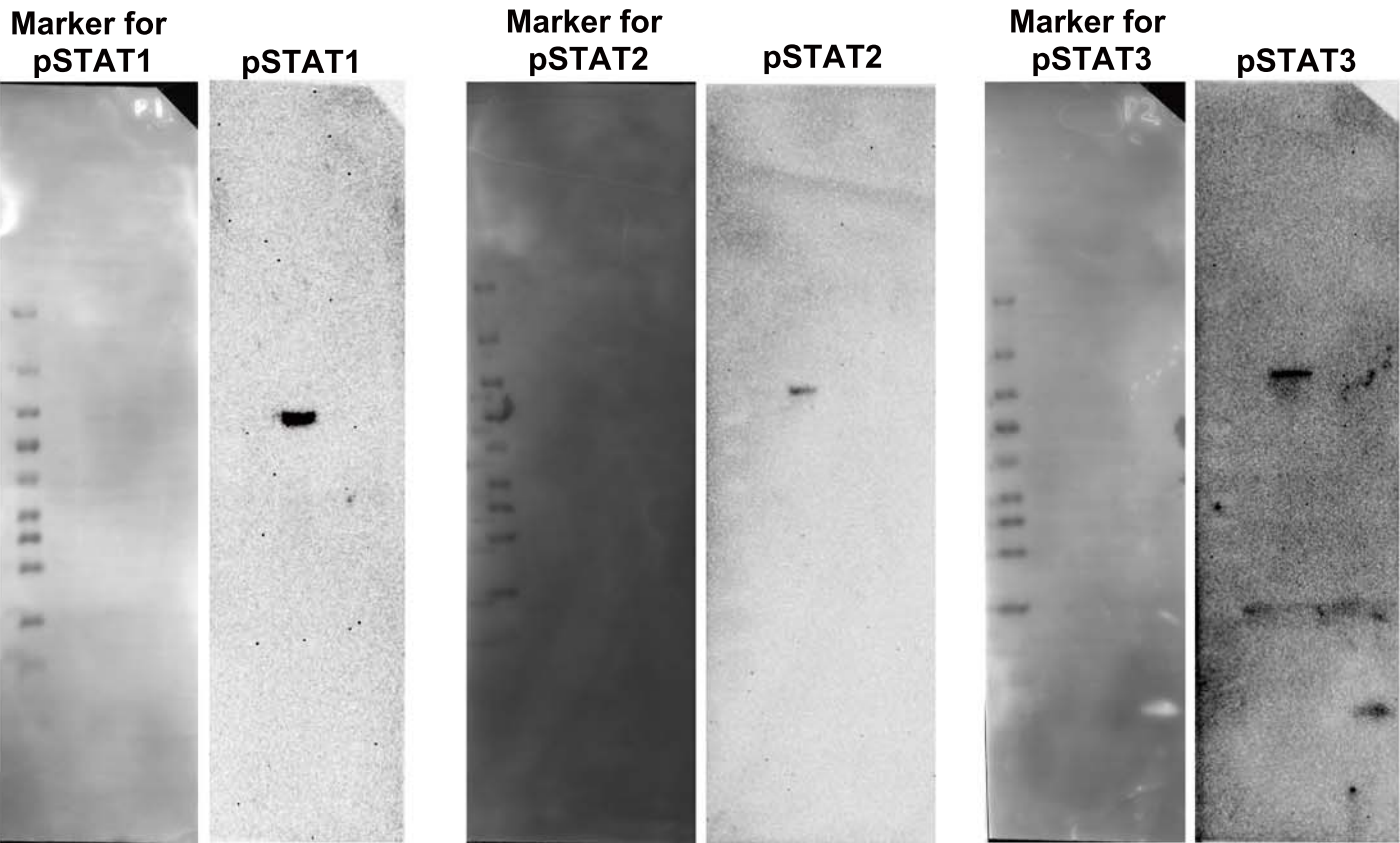

Original Data for Figure 7B

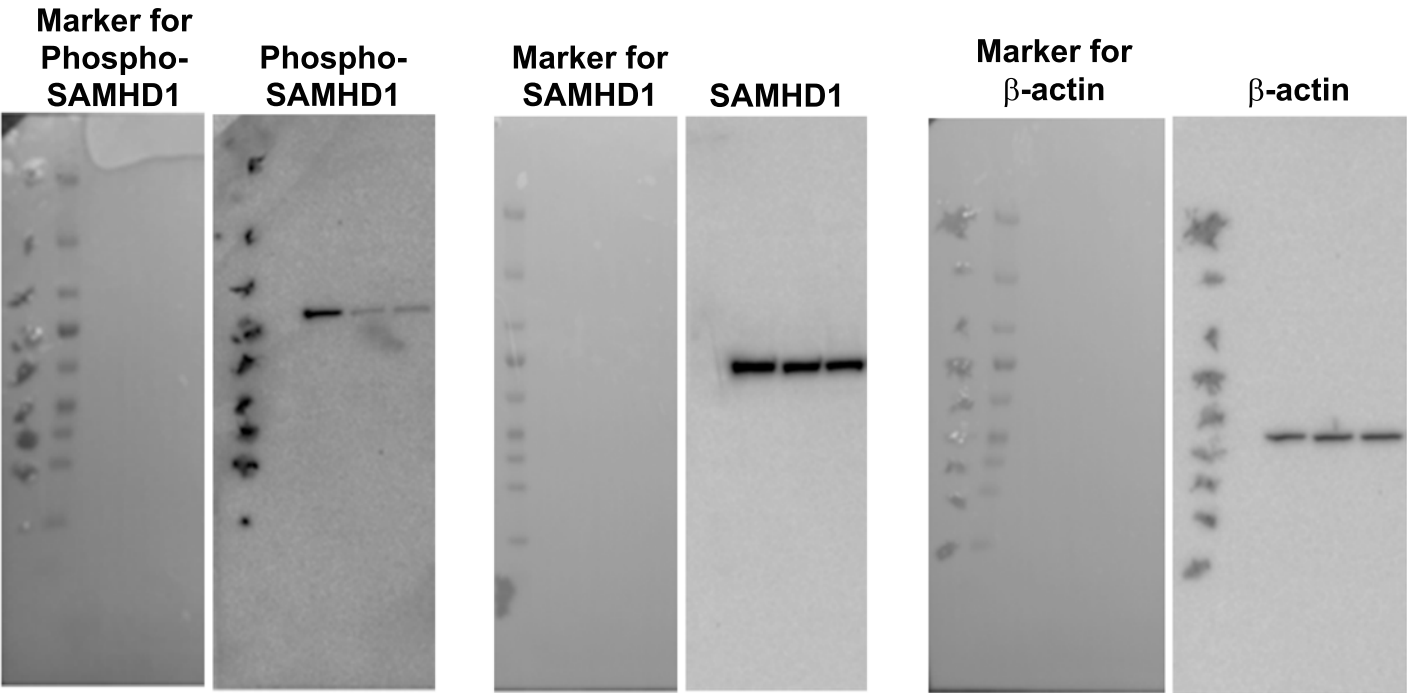

Supplement: Supplementary file 2 — Original Data File [file 41420_2023_1755_MOESM2_ESM.pdf]
